# Effects of nicotine on gastric myoelectrical activity in ICR mice

**DOI:** 10.1186/1750-1326-7-S1-S13

**Published:** 2012-02-07

**Authors:** Eileen Hui-Chuan Wang, Nathalie Percie Du Sert, John Anthony Rudd

**Affiliations:** 1 School of Biomedical Sciences, The Chinese University of Hong Kong, Hong Kong, SAR; 2 Division of Basic Medical Sciences, St. George’s University of London, London, UK

## Background

Slow waves originate from the pacemaker network of the interstitial cells of Cajal (ICC) and determine the direction and velocity of propagation of peristaltic activity of the gastrointestinal (GI) tract. The enteric nervous system (ENS) and smooth muscle cells are known to interface with ICCs through excitatory and inhibitory neurotransmitters. Electrogastrography (EGG) can be used to record gastric myoelectrical activity (GMA) and reveals slow wave information, in terms of frequency and power. GMA has been studied in conscious rabbits, dogs, rats, and ferrets, but rarely in mice. Nicotine was used to activate ganglia in an attempt to modulate excitatory and inhibitory neurons linked to ICC functioning. The aim of the present study, therefore, was to define the characteristics of GMA in mice, and to establish criteria for analysis.

## Methods

16 male ICR mice (3 month old, 28-38 g) were anaesthetized and surgically implanted with telemetry devices (PhysioTel^®^ ETA-F20, DSI) with recording wires sutured from the serosal surface of the stomach. 7 days later, baseline GMA recordings were obtained 2 h before injecting animals with nicotine (3 mg/kg, i.p.; n=8), or vehicle (saline 2ml/kg, i.p.; n=8). Recordings then continued for a further 6 h. Raw data (sampled at 1000 Hz) were analyzed using Spike2 (Cambridge Electronic Design, U.K.). A low pass FIR filter was applied (2.5Hz; transition gap: 10) to eliminate cardiac/respiratory signals. The waveform was then interpolated to 10.24 Hz before applying the second low pass FIR filter (0.5 Hz; transition gap: 0.1). The resultant waveform was then processed further by Fast Fourier Transformation (FFT) to yield components of frequency. Two-way repeated measures ANOVA was performed to compare between the 8 vehicle- and 8 drug-treatment mice. A value of P<0.05 was considered statistically significant.

## Results

The dominant frequency (DF) of the baseline recordings of the vehicle and nicotine treatment groups were 6.8 ± 0.4 and 6.6 ± 0.4 counts per min (cpm), respectively. For the baseline recording of the vehicle and nicotine treatment groups, 40.9-41.8 % of the power was in the normogastric range (DF ± 2 cpm). 9.1-9.7 % and 26.6-26.6 % of the power was in the bradygastric (0 to DF-2 cpm) and tachygastric ranges (DF+2 to 15 cpm), respectively. Saline had no effect on slow waves during the experiment (n=8; P>0.05). Nicotine reduced the DF almost immediately to 5.9 ± 0.5 cpm (n=8; P<0.001) and produced a no-significant increase in % power of bradygastria (P>0.05). The effects of nicotine lasted for 2 h before the DF shifted back to pre-nicotine levels (6.8 ± 0.4 cpm). The % power in tachygastria (DF+2 to 15 cpm) was not affected by nicotine (P>0.05).

## Conclusion

Nicotine, which is known to stimulate ganglia, caused bradygastria, suggesting an action to release inhibitory mediators to affect ICC. The studies demonstrate that radiotelemetry can be used to record GMA in conscious, freely moving mice, providing a convenient method to study GI functioning in a variety of circumstances. The studies were supported by a Direct Grant for Research (2006.2.034).

